# Growth and Antioxidant-Related Effects of the Reestablished Ascorbic Acid Pathway in Zebrafish (*Danio rerio*) by Genomic Integration of L-Gulonolactone Oxidase From Cloudy Catshark (*Scyliorhinus torazame*)

**DOI:** 10.3389/fphys.2021.685595

**Published:** 2021-07-05

**Authors:** K. A. S. N. Shanaka, Sumi Jung, N. D. Janson, J. R. P. Jayasingha, K. P. Madushani, Myoung-Jin Kim, Jehee Lee

**Affiliations:** ^1^Department of Marine Life Sciences & Fish Vaccine Research Center, Jeju National University, Jeju, South Korea; ^2^Marine Science Institute, Jeju National University, Jeju, South Korea

**Keywords:** transgenic zebrafish, ascorbic acid, gene expression pattern, antioxidant genes, morphology

## Abstract

Loss of L-gulonolactone oxidase (*GULO*), which catalyzes the last step of the ascorbic acid (AA) biosynthesis pathway, results in a complete lack of AA in several Osteichthyes fish species, including zebrafish. In this study, *sGULO*, the active *GULO* gene from cloudy catshark (*Scyliorhinus torazame*) was cloned into zebrafish using the Gateway cloning method. The resulting *Tg(b-actin:sGULO:mCherry)* fish were analyzed for the effects of a reestablished AA pathway. Fluorescent microscopy and PCR were used to analyze the integration of the construct into the zebrafish genome. Catalytic activity of sGULO, AA production, growth-related characteristics, and gene expression were investigated to evaluate the effects of AA production in *Tg* fish. The mCherry fluorescent protein indicated the proper integration and expression of the *sGULO* construct in zebrafish. The *sGULO* gene was ubiquitously expressed in all the studied tissues and the enzyme activity indicated an increased AA production in *Tg* fish. The growth of *Tg* fish was also increased, and antioxidant system analysis suggests that reactive oxygen species production was reduced in *Tg* fish compared with wild type. Expression of the AA transporter *slc23a1* was significantly downregulated in *Tg* homozygous fish. These results collectively indicate the effects of reestablished AA synthesis in zebrafish.

## Introduction

L-gulonolactone oxidase (GULO) is the last enzyme in the ascorbic acid (AA or vitamin C) pathway. Almost all plants and a majority of animals have an active GULO enzyme that catalyzes AA synthesis from glucose (Smirnoff, 2001). Prokaryotes lack this ability (Smirnoff, 2001), and thus, obtaining AA through symbiotic relationships from prokaryotes is not an option for the animals who are unable to produce AA. Therefore, animals who do not have an active GULO enzyme must obtain AA from their diet.

The role of AA in animals is more complicated than initially thought (Smirnoff, 2018). The basic function of AA is to act as an antioxidant (Cort, 1982), which is consistent with the finding that most plants and algae produce AA to scavenge the reactive oxygen species (ROS) produced as a byproduct of photosynthesis (Gallie, 2013). In mammals, at least 15 enzymes belonging to the class of oxygenases and amino acid oxidases were found to use AA as an electron donor (Padayatty and Levine, 2016). Additionally, AA has a regulatory role during development (Gallie, 2013) and reported immune defense functions in animals (Carr and Maggini, 2017).

It was believed that all fish species are unable to synthesize AA; however, a later discovery shows that several cartilaginous and non-teleost bony fish species (Coelacanths) are capably synthesizing AA (Drouin et al., 2011). The AA synthesized by cartilaginous fish confirms that a common ancestor of all fish species could produce AA. Interestingly, this capability was later lost in the non-cartilaginous teleost fish, including zebrafish. Furthermore, the reappearance of AA synthesis in coelacanths and amphibians suggests the expansion of AA synthesis into terrestrial vertebrates (Drouin et al., 2011).

The AA synthesis in fish resembles that of terrestrial animals, with D-glucose-1-phosphate as the first precursor of the pathway. An intermediate compound, L-gulonate, can be transformed into L-gulono-1,4-lactone by gluconolactonase. L-gulono-1,4-lactone is then converted into L-AA by a single enzyme, GULO. L-gulonate participates in the pentose synthesis pathway. Therefore, the pathway up until the production of the L-gulonate is conserved in virtually all animal species (Crawford, 1982). Availability of gluconolactonase has been observed in several teleosts, including zebrafish (Cho et al., 2007). However, in animals that cannot produce AA, GULO is non-functional or missing (Drouin et al., 2011).

This study aimed to reestablish the lost AA pathway in zebrafish as a model fish and examine the impact of its reactivation. The active GULO gene from the cloudy catshark (*Scyliorhinus torazame*) (sGULO) was integrated into the zebrafish system by homologous recombination. *In vivo* production of AA and catalytic activity of the sGULO enzyme were confirmed in zebrafish. Physiological and growth-related effects of *Tg* fish were compared with wild-type (Wt) fish along with their gene expression patterns.

## Materials and Methods

### Zebrafish Husbandry

Adult zebrafish were maintained according to standard protocols previously established (Avdesh et al., 2012). Briefly, the fish were grown in a 14:10 h light/dark cycle and a constant water temperature of 28°C ± 0.5°C (pH 6.8–7.5). For the growth assay, fish were fed a meal with or without AA three times (Table 1). During the growth and gene expression assays, the water condition was kept constant in all fish tanks. The animal study was reviewed and approved by the Jeju National University Animal Ethics Committee.

**TABLE 1:** Composition of the diet used for this study.

	AA + (500 mg Kg^–1^) diet		AA- diet
		
Component	Amount (g)/100 g	Amount (g) for 500 g	Amount (g) for 500 g
Wheat gluten^1^	15	75	75
Casein^1^	30.5	152.5	152.5
Egg whites	4	20	20
Cellulose^1^	3	15	15
Vitamin mix (without AA)^1^	4	20	20
Mineral mix^1^	4	20	20
Starch^2^	26.5	132.5	132.5
Corn oil^1^	7	35	35
Soy lecithin^1^	5	25	25
Stay C (AA source)^2^	50 mg	250 mg	0
alpha tocopherol (Vit E)^2^	0.05	0.25	0.25

*^1^https://dyets.com/. ^2^Thermo Fisher Scientific, United States.*

### Bioinformatics Characterization and Isolation of Cloudy Catshark *sGULO*

The nucleotide sequences for *sGULO* and gluconolactonase orthologous were obtained from the National Center for Biotechnology Information (NCBI) (Altschul et al., 1990). The open reading frames (ORFs), putative amino acid sequences, and multiple sequence alignments of *sGULO* and gluconolactonase were analyzed using Clustal Omega (Sievers et al., 2011). A homology model for gluconolactonase was constructed with SWISS-MODEL (Waterhouse et al., 2018), and the PyMOL v2.2.3 software was used to visualize its 3-D structure.

Cloudy catsharks (*Scyliorhinus torazame*) were obtained from the local fish market in Hamdeok (Jeju, South Korea). Fish was dissected to extract the kidneys, which were immediately frozen in liquid nitrogen. The total RNA was extracted from the tissues using Trizol Reagent^®^ (Invitrogen, California, United States). The purity and the concentration were determined using Multiskan GO microplate spectrophotometer (Thermo Scientific, Waltham, MA, United States). The extracted RNA was used to synthesize cDNA using the PrimeScript^TM^ first-strand cDNA synthesis kit (TaKaRa, Kyoto, Japan) following the manufacturer’s instructions.

### Assembling the Expression Construct

We used the Tol2kit to prepare the *b-actin:sGULO:mCherry* construct (Kwan et al., 2007). The primer design and vector selection were made using the Multisite Gateway^®^ three-fragment vector construction kit (Sasaki et al., 2004). Primers for Gateway cloning were designed according to the manufacturer’s instructions (Invitrogen, California, United States). Additionally, the “Kozak” consensus sequence was added to the forward primers. For analysis of differential gene expression, including that of *cat*, *sod1*, *sod2*, *cyb5a*, *procollagen*, *slc23a1*, and *slc23a2*, separate qPCR primers were designed (Supplementary Table 1).

The Tol2kit gateway-based technique was used for all cloning. To prepare the expression construct, pDONR221, p5E-b-actin, p3E-mCherrypA, and pDestTol2pA vectors (Invitrogen, United States) were used in this study.

*sGULO* was amplified using sGULOattB-F/R primers that included the specific attB-cloning sequences for the Gateway vectors (Supplementary Table 1). The PCR products were confirmed by gel electrophoresis and purified using an Accuprep^®^ gel purification kit (BIONEER, Korea). The entry clone was constructed in a multisite Gateway cloning BP recombination reaction according to the manufacturer’s instruction with some modifications (Invitrogen, United States). The constructed entry clone was transformed into One Shot^®^ Top10 chemically competent *E. coli* cells (Promega, Madison, WI, United States). Entry clone plasmids were extracted using an AccuPrep^®^ plasmid mini extraction kit (BIONEER, Korea). Sequences of the entry vector were confirmed before preparing the expression construct.

Expression clones were constructed combining the b-actin promoter, *sGULO* ORF, and *mCherry* reporter gene, respectively. The Gateway LR recombination reaction was employed to prepare *Tg(b-actin:sGULO:mCherry)*. This construct was then transformed into competent *E. coli* using a heat-shock transformation protocol provided by the manufacturer. The plasmid was extracted from positive clones using the QIAGEN^®^ plasmid purification kit (Qiagen, Hilden, Germany).

The pCS2FA-transposase plasmid was linearized by *Not*I (New England Biolabs, Ipswich, MA, United States) and purified with the Accuprep^®^ PCR purification kit (BIONEER, Korea). Linearized DNA (2 μg) was used for the *in vitro* transcription with the mMESSAGE mMACHINE^TM^ SP6 transcription kit (Invitrogen, California, United States). Transcribed RNA was purified using the ethanol precipitation method. RNA concentration was quantified using a Multiskan-GO microplate spectrophotometer (Thermo Scientific, Waltham, MA, United States) and stored at −80°C until needed.

### Construction of Transgenic Zebrafish

A pneumatic pico pump PV830 (World Precision Instruments, Sarasota, FL, United States) was used for the microinjection protocol. The microinjector was calibrated using the standard oil drop method (i.e., measuring the oil drop diameter). The microinjection mixture was prepared by mixing the expression clone, purified transposase mRNA, 0.1 M KCl, and 0.05% phenol red in nuclease-free water (final mixture volume = 10 μL). Prepared one-cell embryos were microinjected with 75 pg expression clone and 25 pg transposase mRNA. The injected embryos were placed in E3 medium and incubated at 28°C.

### Establishment of Transgenic Zebrafish

After 72 h postfertilization (hpf), the microinjected embryos were selected under a fluorescent microscope (DM600B, Leica, Wetzlar, Germany). The embryos that expressed red fluorescence were selected and raised at 28°C. To select the germline transmission in transgenic F0 zebrafish, we crossed injected fish with Wt fish and collected the embryos. After 72 hpf, embryos were checked for red fluorescence. The injected zebrafish that produced the positive embryos were selected as the germline transmitting F0 population.

Fins of *Tg* F1 fish were clipped and individually mixed with 50 μL lysis buffer (10 mM Tris pH 8.0, 0.5 M KCl, 150 μL Tween-20, and 150 μL TritonX-100 in 50 mL DEPC water) supplemented with 1 μL proteinase-K. The mixture was incubated at 60°C for 1 h. Proteinase-K was deactivated by incubating the samples at 95°C for 10 min. The extracted genomic DNA was used as a template for PCR with sGULOattB-F/R primers.

The transgenic F1 embryos obtained from F0 fish were selected under a fluorescent microscope (DM600B, Leica, Germany), nurtured, and raised to adulthood. After individuals were genotyped using PCR, F1 fish were crossed to obtain the F2 generation. The F2 generation was evaluated for fluorescence intensity and sorted into different F2 transgenic lines for *Tg(b-actin:sGULO:mCherry)*, which were maintained as breeding populations.

### GULO Assay and AA Quantification

The sGULO enzyme activity and the endogenous AA were assessed with a standard GULO activity assessment (Ayaz et al., 1976) with some modifications. Briefly, a standard curve for the ascorbate concentration was plotted using an AA standard dilution series. Then, three zebrafish *Tg* and Wt groups were separately weighed (five fish in each group). Whole fish were homogenized in 5 mL 0.05 M sodium phosphate buffer (pH = 7.4, 0.2% deoxycholate), and the homogenates were centrifuged for 30 min at 4°C at 20,000 × *g* in an Avanti^®^ J-E high-speed centrifuge (Beckman Colter Inc., Brea, CA, United States). Then, 4 mL supernatant was added to 5.6 mM L-gulonolactone (Sigma-Aldrich, St. Louis, MO, United States) and incubated at 25°C for 30 min in normal atmospheric conditions (1 atm). The reaction was stopped by adding 2 mL of stopping solution, which contained 18% metaphosphoric acid and 16% trichloroacetic acid (Sigma-Aldrich, United States). Then, 0.1 g acid-washed charcoal (Sigma-Aldrich, United States) was added to the mixture and filtered (570 mm filter paper, Whatman). Four milliliters of the filtrate were added to 1 mL of 2,4-Dinitrophenylhydrazine reagent (Sigma-Aldrich, United States). The test tube was covered with aluminum foil and incubated at 47°C for 90 min before the samples were cooled by swirling them in an ice bath with drop-wise addition of 5 mL 85% H_2_SO_4_ (Sigma-Aldrich, United States). The mixture was incubated for 20 min at room temperature, and the absorbance was measured at 524 nm using the Wt sample as the blank. The activity was calculated using the AA standard curve.

### Feed Preparation

Feed for the growth assay was prepared by mixing the components listed in Table 1. Ingredients were mixed and pelleted using an industrial pelletizer (SP-50, Gum Gang Engineering, Daegu, Korea). Prepared feed was stored at −20°C and manually ground into fine particles with mortar and pestle before feeding.

### Growth Assay

Observable physiological differences were analyzed in *Tg* and Wt larvae at 24, 48, 72, and 96 hpf under a stereo light microscope (Zeiss, Oberkochen, Germany). *Tg(b-actin:sGULO:mCherry)* F1 female and male fish were crossed, and the F2 embryos were collected. For the growth assay, 3 days postfertilization (dpf), embryos were sorted into Wt, heterozygous (He), and homozygous (Ho) groups using fluorescent intensity in the head and trunk-tail regions. From 8 dpf, both *Tg* and Wt fish were fed a diet free of AA (AA−) or with AA (AA+). Fish were anesthetized after 20 days, and the body length was measured using a stereomicroscope (ZEISS, Germany). To check the growth of the fish after 1 month, fish were collected at 30 dpf and fed the AA + or AA− diet for up to 60 dpf. Fish were photographed (ZEISS, Germany) and body length measured using an image analyzing software (Digimizer Version 5.4.4).

### Gene Expression Analysis

Gene expression of *sGULO*, superoxide dismutase-1 and -2 (*sod-1,2*), catalase (*cat*), cytochrome 5b (*cyb5*), procollagen, and solute carrier family 23 member-1 and 2 (*slc23a1*, *slc23a2*) were assessed in 3 dpf F2 fish using qPCR. The qPCR analysis was carried out using a thermal cycler (model Dice TP850, TaKaRa, Kyoto, Japan) (Supplementary Table 1). The transcription levels were analyzed using the Livak method (Livak and Schmittgen, 2001) and the zebrafish elongation factor 1a (*ef1a*) was used as an internal reference (Gene bank ID: AM422110). The transcriptional data were presented as fold expression relative to *ef1a*.

### Statistical Analysis

All experiments were performed in triplicate. For the AA acid quantification assay, a *t*-test was used to determine the statistical significance at *p* < 0.05. For the growth analysis and gene expression experiments, an ANOVA was used with Tukey’s comparison (*p* < 0.05). Body length differences (ΔL) were calculated by subtracting the final length from the initial length.

## Results

### Bioinformatics Analysis

**FIGURE 1:**
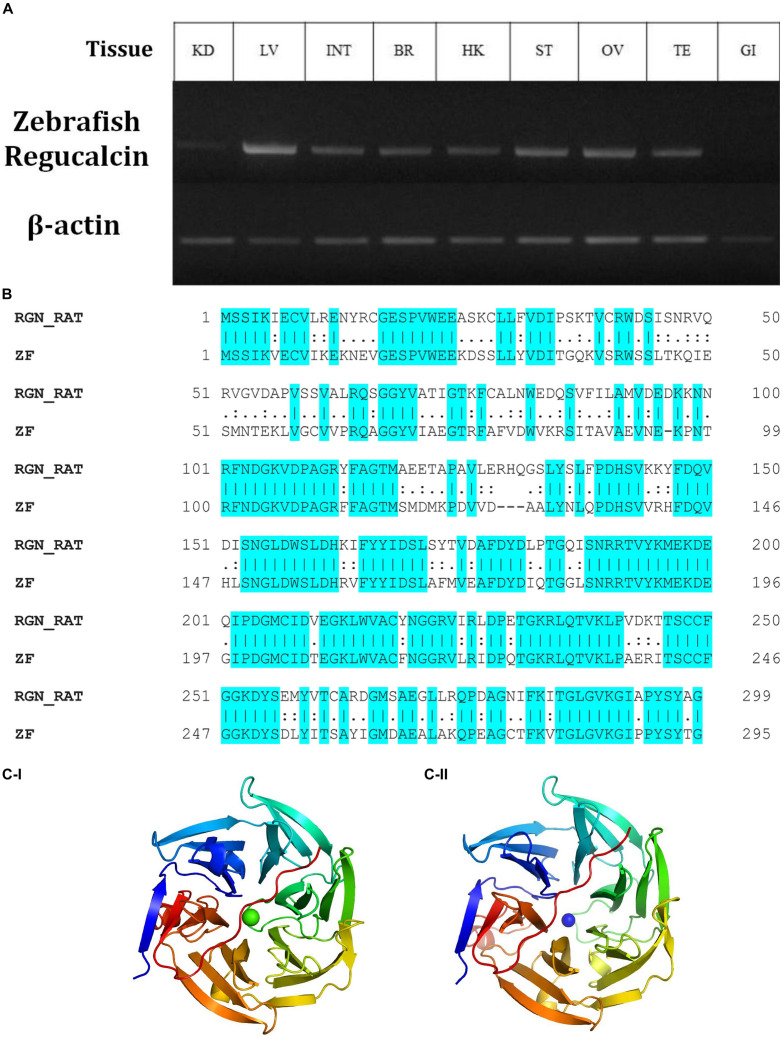
The relative expression of gulonolactonase (*Danio rerio)* in different tissues and its bioinformatics analysis**. (A)** Expression of zebrafish gulonolactonase (regucalcin) in KD (kidney), LV (liver), INT (intestine), BR (brain), HK (head-kidney), ST (stomach), OV (ovary), TE (testis), and GI (gill). Zebrafish β-actin (NM_181601.5) was used as the reference gene for RT-PCR analysis. **(B)** The pairwise sequence alignment between rat (RGN_RAT) (CAA48786.1) and zebrafish (ZF) (NM_205746) gulonolactonase proteins. This alignment indicates high sequence conservation between the rat and zebrafish gulonolactonase proteins. Homology structures for **(C**-**I)** Rat and (**C**-**II)** Zebrafish gulonolactonase. In both structures, the arrangement of the amino acids is similar, indicating conserved catalytic functions.

Gluconolactonase (NCBI accession number, NM_205746) is responsible for the production of L-gulono-1,4-lactone (PubChem CID, 439373), which is the immediate substrate for the GULO enzyme. Therefore, the expression of an active gluconolactonase enzyme in Wt fish is vital for the reestablishment of the AA synthesis in zebrafish. According to the results of this study, Wt zebrafish tissues expressed this enzyme with the highest expression detected in the zebrafish liver, and the other organs tested, except gills, showed more ubiquitous expression patterns (Figure 1A).

Then, the catalytic activity of rat senescence marker protein-30 (SMP30) (CAA48786.1), which is a known protein with gluconolactonase catalytic activity, was compared with zebrafish gluconolactonase (Kondo et al., 2006). Indeed, a pairwise sequence alignment of the zebrafish gluconolactonase with rat SMP30 showed 62.2% identity and 78.6% similarity (Figure 1B). These bioinformatics results further suggest the expression of active gluconolactonase and production of L-gulono-1,4-lactone in zebrafish. Additionally, we constructed separate homology models for both rat (Figure 1C-I) and zebrafish (Figure 1C-II) gluconolactonase enzymes and compared the protein structure; the results indicate a close structural similarity between the two proteins.

### *Tg(b-actin:sGULO:mCherry)* F0, F1, and F2 Generations

**FIGURE 2:**
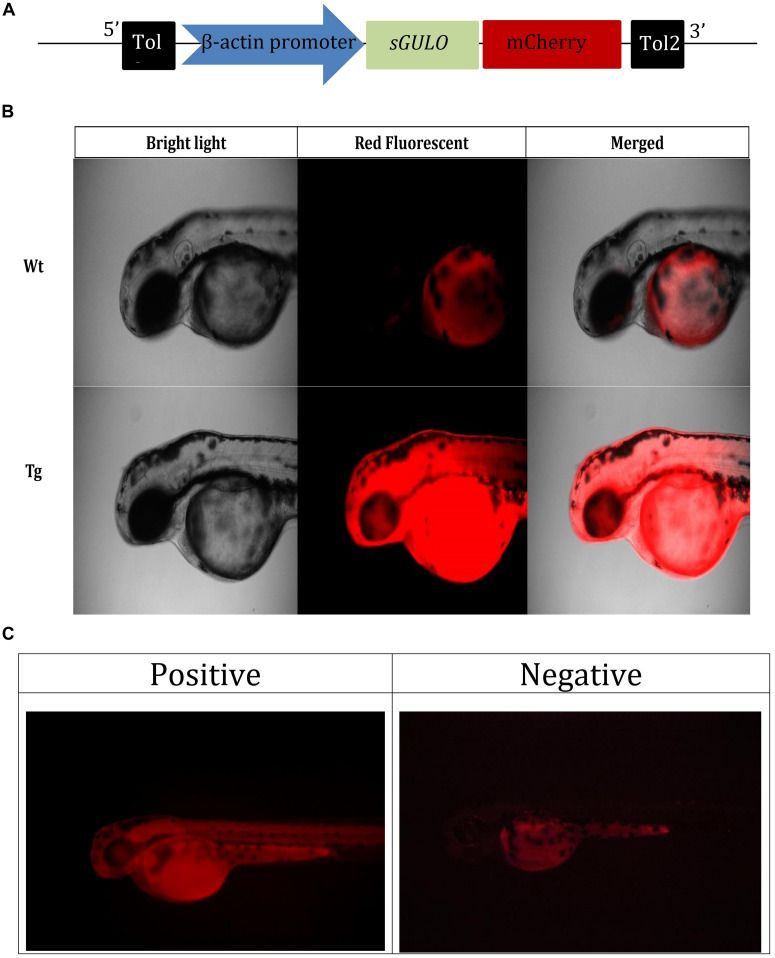
Selection of *Tg* fish expressing *b-actin:sGULO:mCherry***. (A)** A schematic of the *b-actin:sGULO:mCherry* expression construct. The β-actin promoter sequence was used to drive the expression of *sGULO* fused with *mCherry*, which acts as a reporter for construct expression in *Tg* zebrafish. The poly-A segment includes a -G cap mRNA followed by Tol2 sequences, which act as transposable segment sequences needed to integrate the construct into the zebrafish genome. **(B)** The *mCherry* fluorescent signal in *Tg* and Wt fish. **(C)** Positive *Tg* embryos showed red fluorescence in their muscles, and negative fish lacked this fluorescent signal. The positive and negative F0 generation embryos were anesthetized using tricaine, mounted on a microscope slide using 2% methylcellulose, and observed under a fluorescent microscope (Leica, DM600B) under ×100 and ×200 magnifications.

**FIGURE 3:**
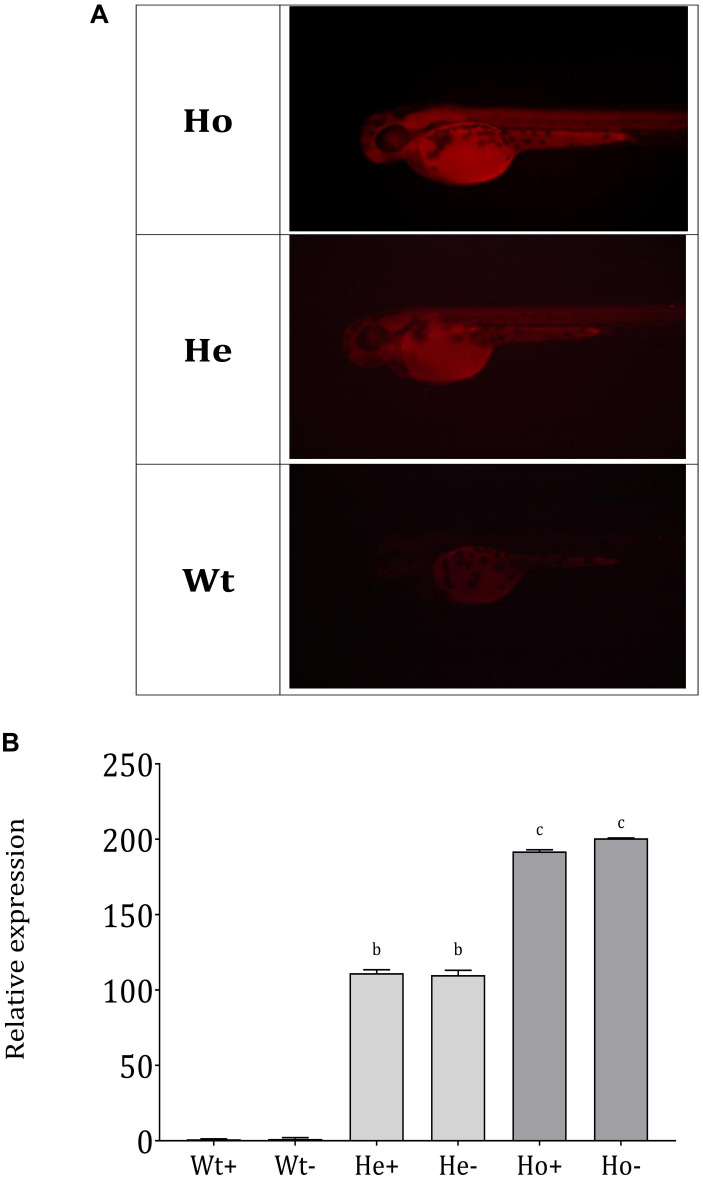
Separation of Ho and He *Tg* fish lineages and *sGULO* expression**. (A)** *Tg* F1 male and F1 female fish were crossed, and the offspring were categorized into three groups based on the fluorescence intensity levels. Wt fish showed no fluorescent signal in their head region and muscles. Ho and He fish had fluorescent signals in their head and muscle regions, which was higher in the Ho fish than in He fish. **(B)** Relative expression of the *sGULO* gene in Wt, He, and Ho fish muscles after 60 dpf. Plus “+” indicates those fed with AA, and the minus “–” indicates those fed without AA. The results were analyzed with ANOVA using Duncan’s test (*p* < 0.05) Different lowercase letters indicate a statistical difference in the mean value (*n* = 10).

We assembled the *Tg(b-actin:sGULO:mCherry)* construct (Figure 2A) for generating *Tg* zebrafish. After the genomic integration, expression of this construct in zebrafish was screened by detecting red fluorescence under a fluorescent microscope (Figures 2B,C). When the fish were 2 months old, they were verified using PCR (Supplementary Figure 1A). Germline transmission of the *b-actin:sGULO:mCherry* construct in the F1 and F2 lines was also analyzed using florescent detection (Supplementary Figure 1B). The F1 generation was reconfirmed with PCR using sGULOattB-F/R primer pair. Ho and He fish in the F2 generation were genotyped using relative fluorescence intensity in their head and muscle regions (Figure 3A). Then, the expression level of *sGULO* was confirmed using qPCR (Figure 3B) to verify fluorescent selection.

### sGULO Enzyme Activity and Endogenous AA Production

**FIGURE 4:**
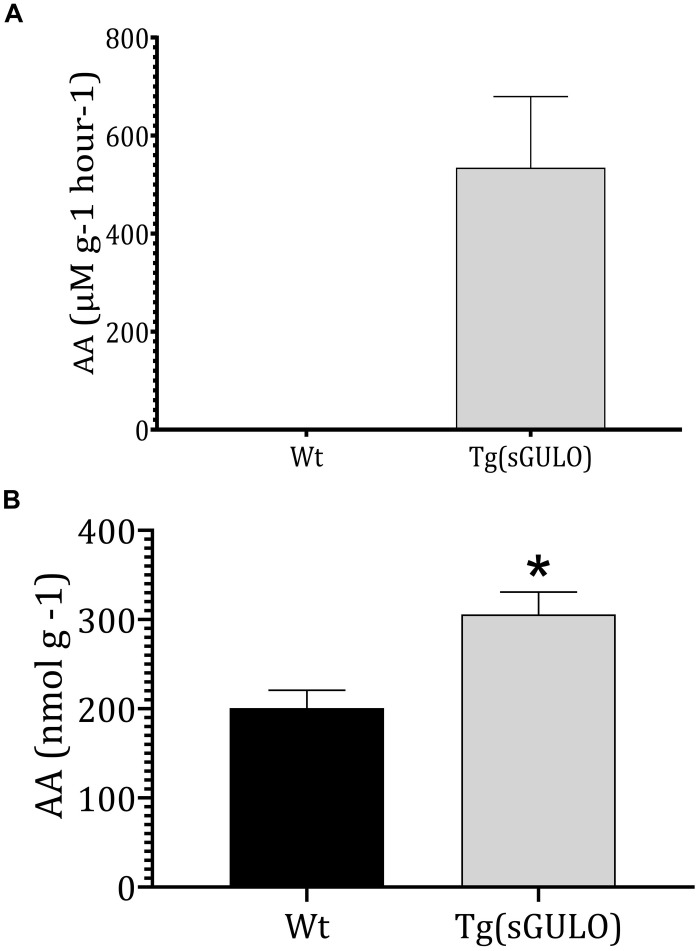
The sGULO enzyme activity and AA level in Wt and *Tg* fish. **(A)** Detection of sGULO activity in *Tg* and Wt fish. Whole zebrafish were homogenized and used to detect the endogenous enzyme activity. This experiment was performed using an Oxi Select AA detection kit. The production rate of AA in Wt and *Tg* fish was measured after the addition of gulonolactone. **(B)** Detection of the endogenous AA level in Wt and *Tg* fish. Whole zebrafish were homogenized and used to detect endogenous AA levels using an Oxi Select AA detection kit. The error bars represent the standard deviation associated with experimental triplicates. The statistical significance between the Wt and *Tg* groups was analyzed using a *t*-test, and the significance is indicated by an asterisk *p < 0.05.

Endogenous sGULO enzyme activity indicated the successful activity of the sGULO enzyme in the zebrafish system. Although GULO activity was absent in Wt fish, *Tg* fish had sGULO activity of more than 534.5 μMg^–1^h^–1^ *in vivo* (Figure 4A). *In vitro* synthesis of AA after the introduction of GULO previously indicated that the mouse GULO could catalyze the formation of AA (Li et al., 2008). Therefore, these results suggest the activity of sGULO enzyme in a *Tg* fish system. A more significant endogenous AA level was observed in *Tg* fish (305 nmol g^–1^) than in Wt (200.8 nmol g^–1^) (Figure 4B). This AA production *in vivo* confirmed the successful reestablishment of the AA pathway in zebrafish. The difference in the amount of the AA produced in *Tg* and Wt fish may provide us a value for the AA solely made by the sGULO, which is 104.2 nmol g^–1^ (305–200.8 nmol g^–1^).

### Physiological Difference

Developmental characteristics between the F2 *Tg* and Wt fish were compared to evaluate the effects of the reintroduced AA synthesis on fish physiology. We found that *Tg* were able to produce AA independent of feeding. Furthermore, endogenous and continuous synthesis of AA may not be identical to the AA provided in the feed meal.

A comparison of the morphology of adult fish shows that the trunk area above the pectoral fin in both male and female *Tg* fish appeared to be muscular compared to Wt fish. As this is an indication of higher muscle mass, we measured the weight of *Tg* and Wt fish separately. *Tg* fish showed significantly higher weight compared to Wt fish (Supplementary Figure 2).

Body shape and external appendages were also compared using a light microscope (Supplementary Figure 3). Results revealed no significant visual abnormalities except the size of *Tg* fish. Activity during feeding and general swimming patterns were also quite similar between *Tg* and Wt fish. Overall, there was a lack of observable behavioral differences between *Tg* and Wt fish, indicating that the expression of *sGULO* transgene and AA synthesis did not cause any physiological abnormalities in *Tg* fish.

Growth analysis was performed to investigate the growth-related effects of reestablished AA synthesis on zebrafish development. During this experiment, growth was analyzed at two stages. Early growth was analyzed during the first 20 days of feeding (8–28 dpf), and latter growth was analyzed during 30 days of feeding (30–60 dpf).

**FIGURE 5:**
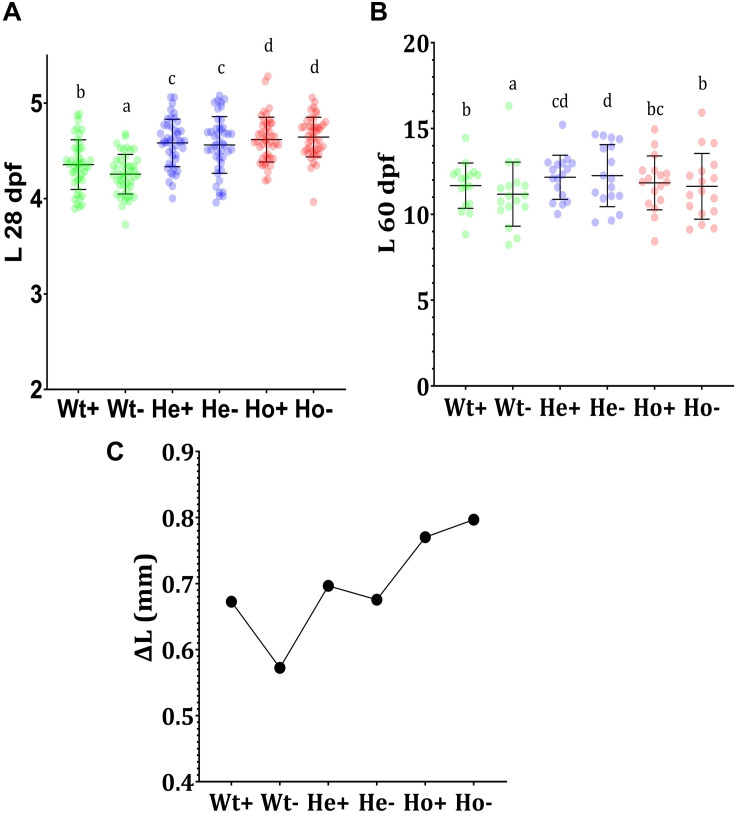
Feeding experiment and evaluation of growth *Tg* (*b-actin:sGULO:mCherry*) fish**. (A)** The length (mm) of Wt and *Tg* fish after the first 20 days of feeding (8–28 dpf), **(B)** The growth of the latter 30 days of feeding (30–60 dpf), and **(C)** the growth difference (ΔL) during the first 20 days of feeding. Every single point overlayed on the charts indicates an individual fish. Error bars represent the standard deviation of the individual body lengths. Data were analyzed with ANOVA using Duncan’s test (*p* < 0.5), and different lowercase letters indicate different significant levels between samples. Plus (+) and minus (-) labels indicate AA plus and absent meals, respectively.

The results of 28 dpf (Figure 5A) showed the effect of AA on development. Significantly lower growth of AA-fed Wt fish (Wt-) compared to AA + fed Wt fish (Wt +) indicates the importance of AA in growth-related activities in zebrafish. Furthermore, Ho fish showed higher body length. In between He + and He- as well as Ho + and Ho-, body length was statistically similar. This may indicate the production of an adequate level of AA in *Tg* fish. According to the length at 60 dpf (Figure 5B), He- fish showed the highest growth; interestingly the growth of Ho + fish was equal to Wt + fish. To clearly understand the rate of growth, we calculated the growth difference graph (Figure 5C). Even though the highest growth rate was expected in the Ho + fish, these results indicate that Ho- fish had the highest growth rate.

The enhanced growth of *Tg* fish when compared with Wt fish may have arisen due to the constant and ubiquitous availability of the reduced form of AA in *Tg* fish. As AA is being generated in the body of *Tg* fish, it is readily available for development-related biochemical activities. During early stages of development, this constant availability of AA may be an important factor for developmental processors; however, the length of *Tg* fish in the latter stages was not significantly different from that of Wt fish fed with the AA + diet.

### Gene Expression Analysis

**FIGURE 6:**
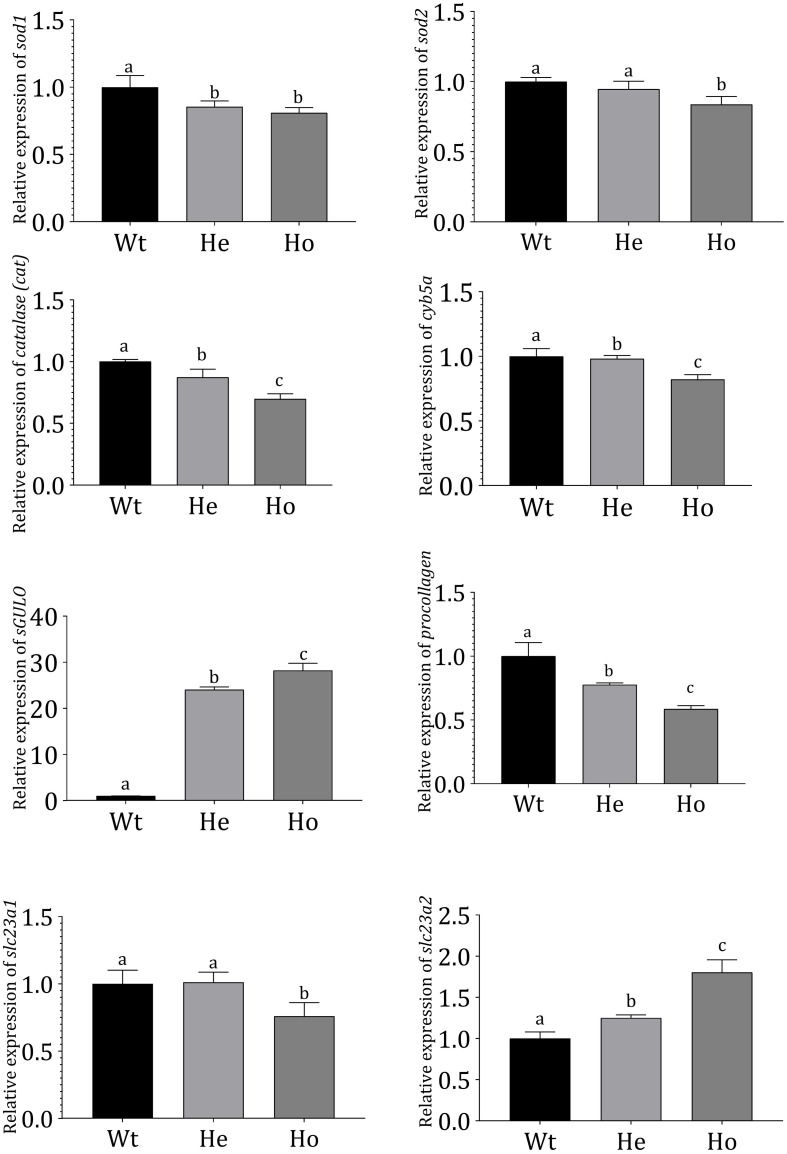
Gene expression profiles in *Tg* and Wt fish. Gene expression profiles of Wt and 3 dpf F2 *Tg* zebrafish (*n* = 50 for each sample). mRNA expression was measured using qRT-PCR and evaluated using the Livak method (Livak and Schmittgen, 2001). *Danio rerio ef1-*α expression was used as an internal control. Error bars represent the standard deviation of triplicate experiments. Data were analyzed with ANOVA using Duncan’s test (*p* < 0.05), and significant differences between *Tg* and Wt groups are indicated by different lowercase letters.

The expression of genes in F2 *Tg* fish was evaluated (Figure 6); superoxide dismutase-1 (*sod1*), superoxide dismutase-2 (*sod2*), catalase (*cat*), and cytochrome b5 type-α (*cyb5a*) expressions were analyzed in *Tg* and Wt zebrafish.

According to our results, *cat*, *cyb5a*, *sod1*, and *sod2* were significantly reduced in *Tg* fish. Notably, the reduction was more prominent in Ho fish compared with He fish. In these *Tg* fish, AA production was expected to reduce ROS. The *sGULO* gene expression analysis showed that the Ho fish had the highest expression of *sGULO*, indicating higher production of AA than the He line. This type of AA production in *Tg* Ho fish could have resulted in the observed reduction of the oxidative system-related genes analyzed. Interestingly, we also observed a significant reduction in *procollagen* gene expression in the presence of high *sGULO* expression.

We analyzed and compared several AA transporters in *Tg* zebrafish to Wt fish to understand the effect of AA to express receptors for AA absorption. According to this experiment, we observed significant downregulation of *slc23a1* in Ho fish although the expression of *slc23a2* was significantly upregulated. This result is crucial to understand the regulation of AA concentration in *Tg* fish.

## Discussion

One studied example for the gluconolactonase activity is rat SMP30, which catalyzes the conversion of L-gulonic acid (PubChem CID 6857417) to L-gulono-1,4-lactone (Kondo et al., 2006). Previous studies report the presence of catalytically active regucalcin in zebrafish tissues (Cho et al., 2007), which is vital for this pathway reconstruction. As the name suggests, regucalcin is involved in calcium homeostasis. Knockout models for this enzyme developed scurvy within a short time period. Later, it was found that regucalcin acts as a gluconolactonase (Fujisawa et al., 2011; Yamaguchi, 2013).

In this study, we demonstrated the capability of AA pathway reconstruction by introducing an active GULO enzyme. Thereby, the AA pathway is shown to be conserved until the production of L-gulono-1,4-lactone in zebrafish. This pathway conservation up to the last enzyme is important because the latter intermediates of the AA pathway are shown to participate in other metabolic activities (Figueroa-Méndez and Rivas-Arancibia, 2015).

The feasibility of AA pathway reestablishment by introducing the last enzyme has been demonstrated earlier in Gulo-/- mouse. This reconstruction is not limited to the vitamin-C pathway; thus, other pathways can be manipulated accordingly. In humans, this type of pathway reconstruction may be important in therapeutic medicine (Li et al., 2008). As mentioned previously, a common ancestor of Chondrichthyes and Osteichthyes fish had an active GULO enzyme, and it later disappeared in most of the Osteichthyes. Importantly, we attempted to obtain an active GULO enzyme from another fish (*S. torazame*), which may resemble the previously lost GULO enzyme in zebrafish.

The first active GULO enzyme in fish was discovered nearly 20 years ago in *S. torazame* using bioinformatics techniques (Cho et al., 2007) and also showed the catalytic activity of the sGULO enzyme *in vivo*. The authors analyzed the GULO enzyme and reported the highest activity in the kidneys. No active gulo enzyme has been detected in zebrafish to date (Ching et al., 2015). As zebrafish is unable to synthesize AA, the requirement for AA must be fulfilled by diet. The dietary supplement of AA in fish meal generally ranges from 25 to ∼2,000 mg Kg^–1^ and is known to improve the quality of fish (Yildirim-Aksoy et al., 2008; Dawood and Koshio, 2018; Li et al., 2020). In a previous study, zebrafish were fed an experimental diet containing 350 mg Kg^–1^ AA, and approximately 100 nmol g^–1^ AA was present in the zebrafish system (Kirkwood et al., 2012). We observed that our *Tg* fish model generated a similar amount of AA in the body.

The AA requirement may be different between fish species. It may also depend on the development stage and physio-pathological conditions of the fish (German Nutrition Society (DGE), 2015; Wang et al., 2017). Regarding AA utilization, studies show that AA is essential for biosynthesis activities, such as collagen synthesis (Murad et al., 1981; Khan et al., 2017). Additionally, AA could directly provide electrons to metabolic reactions (Cathcart, 1991), and therefore, AA is an important cofactor for multiple enzymatic reactions. Generally, metabolic reactions generate free radicals and oxidative species as byproducts. In particular, lipid metabolism increased production of oxidative species (Le Lay et al., 2014). As an antioxidant, AA may facilitate metabolism without oxidative damage from oxidative radicals. Increased AA concentration promotes good health in fish by enhancing immune reactions against pathogens (Carr and Maggini, 2017). Reduced risk of infections generally improves growth. Therefore, we believe that these factors cumulatively enhance the growth patterns observed in *Tg* fish when compared with Wt fish.

AA can function independently or in conjugation with vitamin E (Liu et al., 2019). Indeed, AA shows a separate role as an antioxidant due to its high water solubility compared with vitamin E (Kurutas, 2015). High concentrations of AA reportedly function at the same rate as certain important antioxidant enzymes, such as sod (Smirnoff, 2018). Interestingly, this enhanced antioxidant capacity may retard some physiological processes, such as muscle synthesis (Le Moal et al., 2017). A previous study shows that increased AA concentration enhanced weight gain in yellow catfish [*Pelteobagrus fulvidraco* (Richardson)] but only up to a level. With a higher AA concentration of more than 156 mg Kg^–1^, no significant increment in weight was observed in yellow catfish (Liang et al., 2017). Accordingly, we believe that the 30–60 dpf length of Ho fish is not prominent due to the similar growth retardation effect.

According to the growth results at 60 dpf, external supplementation of AA is enough to facilitate similar growth in Wt fish compared with Ho- fish. As omnivorous fish in their natural environments, zebrafish may be able to obtain sufficient AA from their diet (Ge, 2018). Therefore, zebrafish may be able to dispense gulo enzyme activity and achieve optimum growth with dietary AA (Fracalossi et al., 2001). In contrast, catshark, being a carnivorous fish, has difficulty in obtaining an adequate amount of AA from its diet (Ching et al., 2015). Therefore, it must retain an active GULO enzyme. During evolution, the lack of Gulo enzyme might have been compensated by increasing the expression of AA transporters. Two *slc23a* genes are translated into two sodium-ascorbate co-transporters (SVCT); *slc23a1* translated into svct1 and *slc23a2* translated into svct2. Svct1 is important for AA absorption through the gut and transportation of AA throughout the body. Svct2 is not involved in AA absorption in the gut, and it is mainly involved in AA uptake into the metabolically active tissues. Thus, svct2 reduces oxidative damage in the metabolically active organs (Savini et al., 2008; Duell et al., 2013). Observed downregulation of the *slc23a1* in Ho zebrafish may further clarify the lack of statistical significance in between the *Tg* fish fed with AA + and AA- diet. Increased AA concentration in the tissues may have reduced the expression of *slc23a1*, which negatively influences the AA absorption and transportation throughout the body.

Regarding the antioxidant system, both sod1 and sod2 influence ROS (Nojima et al., 2015). The sod genes convert superoxide free radicals (O_2_^–^) into H_2_O_2_ and O_2_. H_2_O_2_ is later neutralized by the activity of catalase (Alfonso-Prieto et al., 2009). Further, cyb5a can act as an electron carrier for redox reactions, wherein it catalyzes the conversion of methemoglobin to hemoglobin, which aids O_2_ delivery to the liver and muscle tissues. A deficiency of cyb5a can lead to the physiological condition of methemoglobinemia (Siendones et al., 2018).

Ascorbic acid can directly interact with O_2_^–^ or H_2_O_2_ (Pehlivan, 2017), which is consistent with the observed downregulation of *sod1* and *sod2* in *Tg* fish. *Cat* expression was significantly reduced in *Tg* fish compared with Wt fish. As observed for *sod1* and *sod2* expression, *Tg* fish showed signs of reduced ROS. The observed reduction in *cat* expression could be the result of two factors: the reduction in the precursors of H_2_O_2_ generation, such as O_2_^–^ or the direct removal of H_2_O_2_ by AA. Significant downregulation of *cyb5a* indicated a potential reduction in redox reactions in *Tg* fish compared to Wt fish. In plants, electron transport utilizes AA and its functions in photosynthesis reactions are well characterized (Gallie, 2013). This is also true for animals in which AA can carry electrons between different metabolic reactions (Smirnoff, 2018). Therefore, electron carriers could be weakly expressed in the presence of AA and could explain the reduced expression of the electron carrier *cyb5a* in *Tg* fish compared with Wt fish.

Collagen is a structural material for various connective tissues; previous studies indicate the involvement of AA in collagen synthesis (Qiao et al., 2009). Two enzymes in the collagen biosynthesis pathway, lysyl hydroxylase and prolyl hydroxylase, require AA as an essential cofactor for their enzymatic activities (Pinnell, 1985). These studies also indicate that AA is not involved in the procollagen gene expression. Thus, we believe that the sharp reduction of procollagen transcripts of Ho could be attributed to either transcriptional regulation of collagen biosynthesis, or reduced ROS in *Tg* fish may have negatively affected the procollagen mRNA transcription. However, further studies are required to validate this observation.

## Conclusion

In summary, we develop a transgenic zebrafish by integrating the L-gulonolactone oxidase gene from the cloudy catshark into the zebrafish genome, thereby reactivating the evolutionarily lost AA synthesis pathway. The integrated *sGULO*, under the β-actin promoter, was ubiquitously expressed in transgenic zebrafish, and the expression was confirmed using the mCherry fusion protein as the fluorescent reporter. The catalytic activity of sGULO in *Tg* zebrafish was confirmed by analyzing the AA production. *Tg* fish below 1 month of age showed significantly increased length compared with Wt fish. However, Ho *Tg* fish, which were more than 1 month old, did not show a significant increase in length compared with AA-fed Wt fish. Expression analysis of genes related to the antioxidant system indicate a significant reduction in ROS in *Tg* fish compared with Wt fish. The expression of *slc23a* was significantly reduced in *Tg* fish, indicating that *Tg* fish may be less efficient in AA absorption. These results collectively indicate the involvement of AA in the development and antioxidant system and provide a comparison of growth of internally synthesized AA with externally provided AA.

## Data Availability Statement

The original contributions presented in the study are included in the article/Supplementary Material, further inquiries can be directed to the corresponding author/s.

## Ethics Statement

The animal study was reviewed and approved by Animal Experiment Ethics Committee of Jeju National University.

## Author Contributions

KS, SJ, and NJ: data curation, methodology, software, visualization, writing—original draft preparation, conceptualization, and reviewing. JJ: software. KM: writing—original draft preparation, reviewing, and editing. M-JK and JL: investigation, monitoring, reviewing, resources, and supervision. All authors contributed to the article and approved the submitted version.

## Conflict of Interest

The authors declare that the research was conducted in the absence of any commercial or financial relationships that could be construed as a potential conflict of interest.
