# Supplementary material for: Growth and Antioxidant-Related Effects of the Reestablished Ascorbic Acid Pathway in Zebrafish (Danio rerio) by Genomic Integration of L-Gulonolactone Oxidase From Cloudy Catshark (Scyliorhinus torazame)
Source: Front Physiol. 2021 Jul 5;12:685595. doi: 10.3389/fphys.2021.685595 (PMC8287572; doi:10.3389/fphys.2021.685595)
Supplement: Supplementary file 1 [file Table_1.DOCX]

Supplementary Material

# Supplementary Figures and Tables

## Supplementary Tables

# Supplementary Table 1. Primer sequences used for this study.

| Purpose | Name | Details | Primer sequence (5’-3’) |
| --- | --- | --- | --- |
| SGULO Amplifying | sGULOattB-F | *S. torazame* GULO sequence amplification for attB reaction  AY039838 | GGGGACAAGTTTGTACAAAAAAGCAGGCTTCACCATGGATCAAGGCACCATGGGA |
|  | sGULOattB-R |  | GGGGACCACTTTGTACAAGAAAGCTGGGTCGAAAAAGGTCTTTTCCAGGTAATTGTTGAGG |
| Expression  analysis | sGULO | *S. torazame*  GULO-AY039838 | CAGATCGCACCAACAAGCCAATCA |
|  |  |  | AGCAGCCAATAGAAGAGGCGATTGA |
|  | cat | *Danio rerio* Catalase-  BC051626.1 | CTCAGGTGCTAAATGAAGCCGAGAGA |
|  |  |  | AGAGCCTGAACTCGGTTGCCATAA |
|  | sod1 | Superoxide dismutase 1, soluble- NM_131294.1 | AGCCAGTGAAGGTGACTGGTGAAA |
|  |  |  | TATCGGTTGGCCCACCATGAGTTT |
|  | sod2 | Superoxide dismutase 2, mitochondrial-  NM_199976.1 | TCACAGCAAGCACCATGCAACA |
|  |  |  | CACCGCCATTGGGTGACAGATTT |
|  | cyb5a | Cytochrome b5 type A (microsomal) (cyb5a)- NM_213135.2 | TGCCAAACCACCAGAATCCCTTGTA |
|  |  |  | TCAAGGTGACGATTACAGCAGCCA |
|  | procollagen | collagen, type I, alpha 1a (col1a1a) -NM_199214.1 | ACCGATGGCTTCCAGTTCGAGTATG |
|  |  |  | TTGCCAGAAGCCTGGTCCATGTAT |
|  | gluconolactonase | Regucalcin (rgn) NM_205746 | CAGCTGTTGCCGAAGTTAATG |
|  |  |  | ACCAGCCTCAGGTTGTTTAG |
|  | *slc23a1* | solute carrier family 23 member 1  NM_001173499.1 | GACTGCTAGGAACAGGAAAC |
|  |  |  | CCAGCTCCATACTGAATCAC |
|  | *slc23a2* | solute carrier family 23 member 2  XM_021472134.1 | TGGCTCTACATCTTCCAGCCCAAA |
|  |  |  | CAGCGATGCAAACAGAGCGCTAAA |
|  | β-actin | NM_181601.5 | GAGCAAGAGAGGTATCCTGACC |
|  |  |  | CGGAGCTCATTGTAGAAGGTGTG |
|  | ef1-α | L23807 | CTCCTCTTGGTCGCTTTGCT |
|  |  |  | CCGATTTTCTTCTCAACGCTCT |

## Supplementary figures

(A)


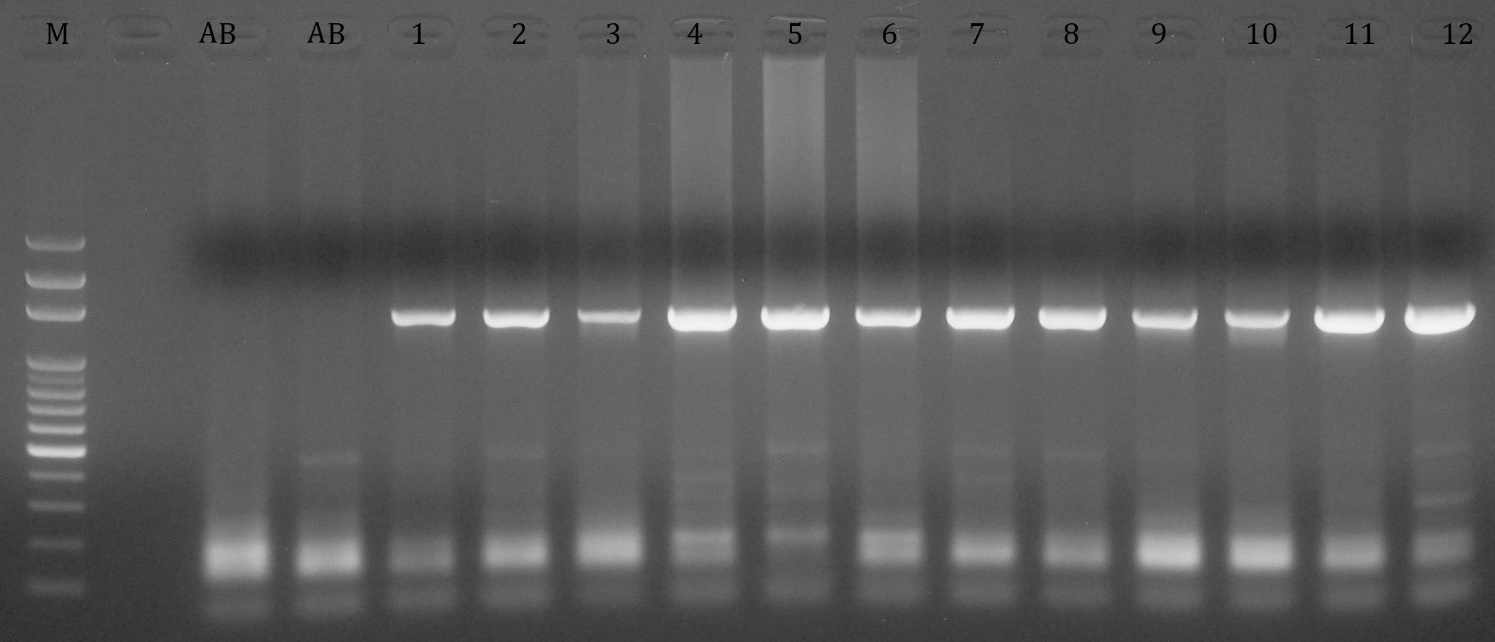


(B)


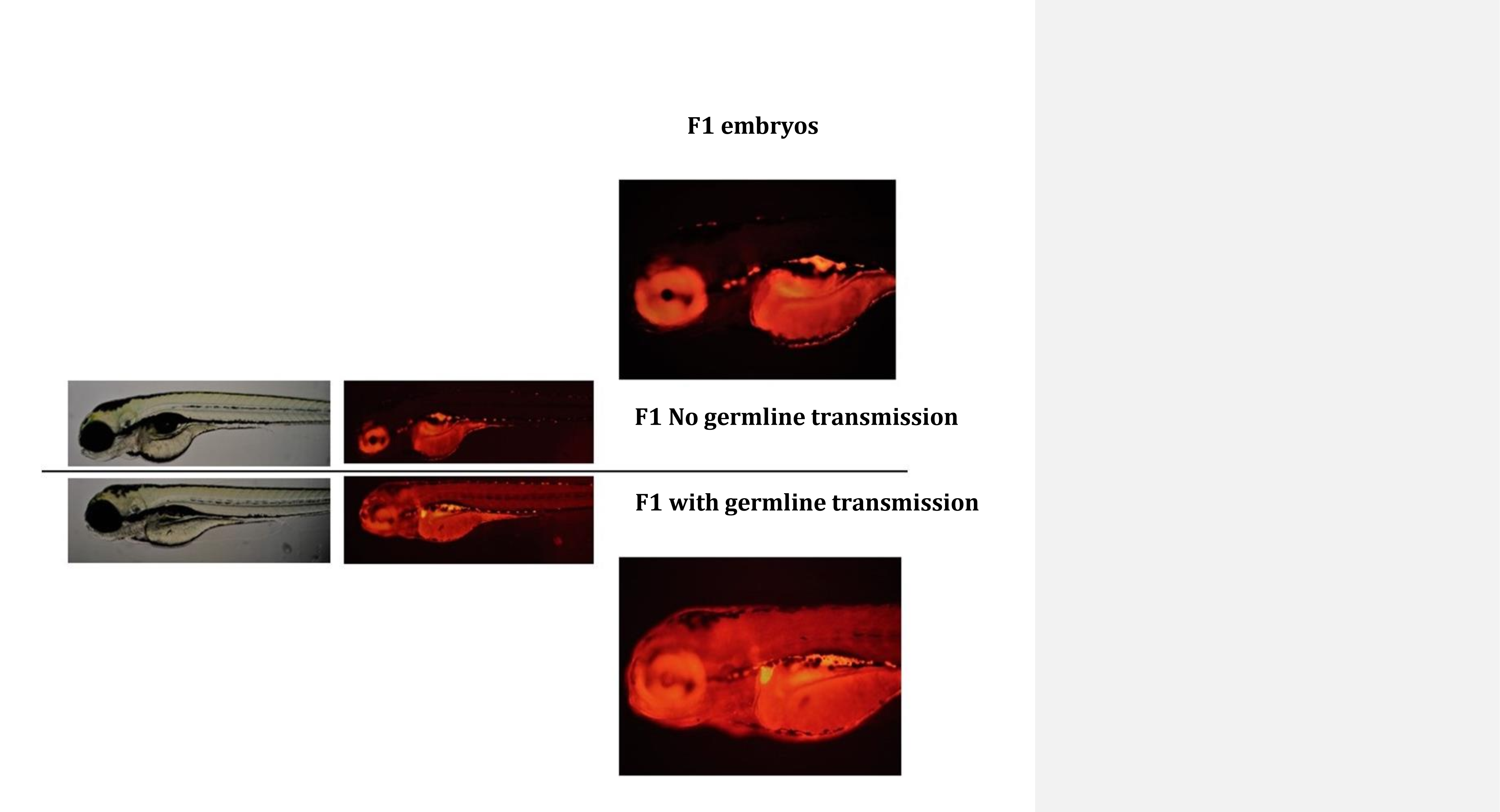
**Supplementary Figure S1.** **Certifying that F1 fish is transferring *sGULO* gene in germ line manner. (A)** The gel image of the *Tg* and Wt zebrafish genotyping**.** The PCR results obtained by the genotyping of transgenic (Tg) and wild type (Wt) zebrafish with M-100 bp marker, 1-12 are the sample numbers used in this experiment. PCR band of size 1320 indicate positive fish. **(B)** Fluorescent evidence for the germ line transfer of the *sGULO* gene to the F1 generation.


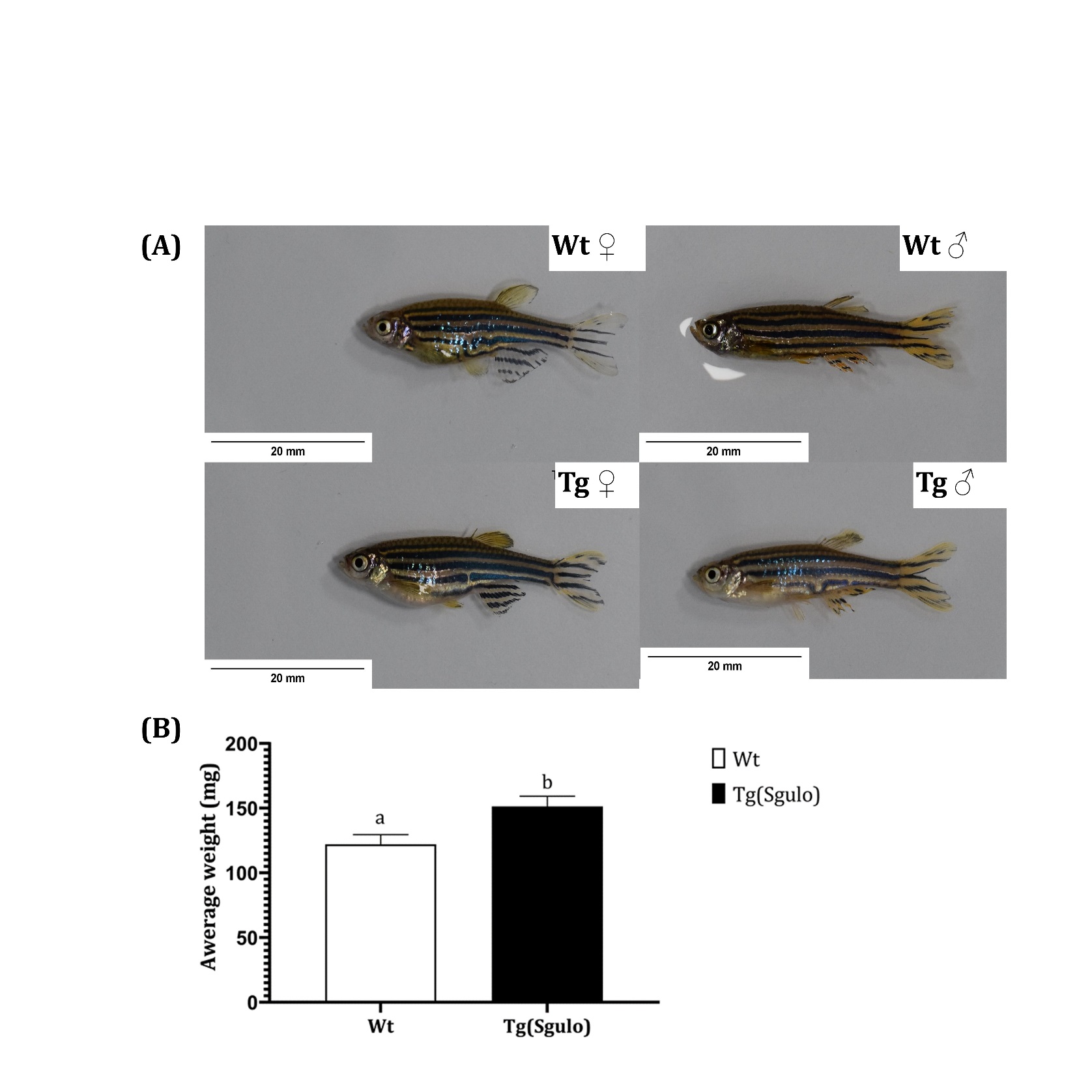


**Supplementary Figure S2. Phenotype of the *Tg*(*β-actin:sGULO:mCherry*) and Wt fish**

**A.** Phenotypic comparison of the Adult *Tg* and Wt fish, *Tg* fish appeared to more muscular compared to Wt fish. **B.** The average growth was analyzed by measuring the total body weight of 3 months old zebrafish (n=10). The error bars represent the standard deviation with experimental replicates. The significant difference between the Wt and *Tg* group is indicated with different lower-case letters (*p*<0.05).


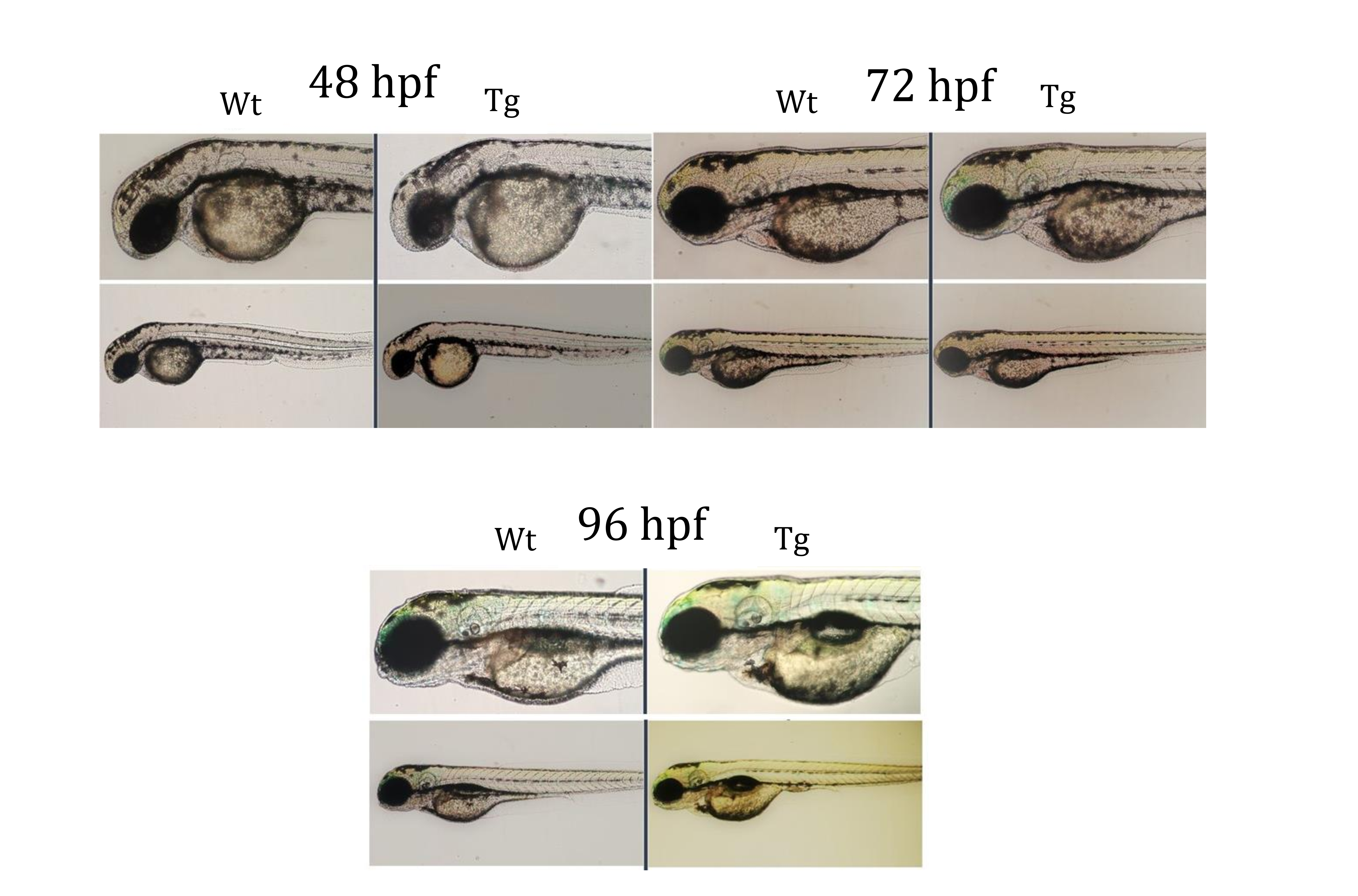


**Supplementary Figure S3. Growth of *Tg* and Wt fish at different developmental stages.** The Physiological differences in the developmental stages of zebrafish at 48, 72, and 96 hpf was compared under the light microscope (Leica, DM600B) at (100× and 200×) magnifications.
